# Transduction of hematopoietic stem and progenitor cells by an *MECP2* lentiviral vector improves Rett syndrome phenotypes

**DOI:** 10.3389/fddsv.2025.1545391

**Published:** 2025-02-20

**Authors:** Joseph S. Anderson, Jill L. Silverman, Alyse L. Lodigiani, Camilla M. Barbaduomo, Julie R. Beegle

**Affiliations:** 1 Department of Internal Medicine, University of California Davis Medical Center, Sacramento, CA, United States; 2 Department of Psychiatry and Behavioral Sciences, University of California Davis Medical Center, Sacramento, CA, United States

**Keywords:** *MECP2* lentiviral vector, Rett syndrome, hematopoietic stem and progenitor cells, gene therapy, neurodevelopmental disorders

## Abstract

**Introduction:**

Rett Syndrome is a genetic neurodevelopmental disorder caused by decreased levels of MeCP2. Due to mutations in the *MECP2* gene, insufficient MeCP2 protein levels lead to clinical phenotypes including the loss of normal movement, decreased communication, seizures, sleep disorders, and breathing problems. Currently there is no cure for Rett Syndrome and the only means to help patients is palliative care directed to their specific symptoms. Therefore, novel therapies need to be developed to alleviate disease phenotypes by restoring normal *MECP2* expression. An autologous hematopoietic stem cell and gene therapy approach for Rett syndrome may offer a benefit to affected patients by systemic delivery of functional MeCP2, including to affected neurons in the central nervous system.

**Methods:**

In our current experiments, we evaluated the therapeutic effect of *MECP2* lentiviral vector transduced human CD34+ hematopoietic stem and progenitor cells after transplantation into an immunodeficient mouse model of Rett syndrome.

**Results:**

We observed improvement of Rett syndrome-related phenotypes including the reversion toward normal motor abilities in an open field assay for total activity, horizontal activity, and vertical rearing activity, and an increased latency to fall in a rotarod assay. An increased level of MeCP2 protein was also observed in the brain tissue of transplanted mice.

**Discussion:**

By providing functional MeCP2 to affected cells, our results highlight the ability of this strategy to improve Rett syndrome phenotypes. These proof-of-concept studies demonstrate the potential use of a stem cell gene therapy approach as a novel treatment for Rett syndrome patients.

## 1 Introduction

Rett Syndrome (RTT) is a progressive X-linked neurodevelopmental disorder (NDD) caused by insufficient levels of the methyl-CpG binding protein 2 (MeCP2) protein due to loss of function mutations in the *MECP2* gene. MeCP2 is a nuclear-specific protein required to bind methylated DNA to regulate gene expression and is expressed in nearly all cells (Zhou and Connolly, 2019). Mutations in *MECP2* result in random X chromosome inactivation, thus mainly affecting females, yet with high variability in phenotypic severity since RTT females are mosaic, with cells expressing either the mutant or wild-type allele of *MECP2*. MeCP2 serves a key function in processes such as: transcriptional repression of methylated genes, the structure of heterochromatin, chromatin looping splicing, activation-dependent regulation of transcription, modulation of long-range genes, and even potentially direct promoter activation (Nan and Bird, 2001; Nan X. et al., 1997; Georgel PT. et al., 2003; Horike S. et al., 2005; Young and Zoghbi, 2004; Zhou G. L. et al., 2006; Zhou H. et al., 2006; Yasui DH. et al., 2007; Chahrour M. et al., 2008).

Numerous genes dependent on MeCP2-mediated regulation have been discovered and it has been demonstrated that disregulation of these genes due to decreased *MECP2* expression leads to the phenotypes associated with Rett syndrome (Ben-Shachar S. et al., 2009; Gabel HW. et al., 2015; Chen L. et al., 2015). Characteristics of RTT include profound intellectual disability, lack of speech, disabilities in mobility, impaired gait and ataxia, loss of purposeful yet frequent hand movements, respiratory issues, and seizures (Na and Monteggia, 2011; Kudo S. et al., 2001; Ip et al., 2018). RTT individuals have poor sleep quality in slow wave and REM stages and obstructive respiratory apnea events are frequent and recurrent (Na and Monteggia, 2011; Kudo S. et al., 2001; Ip et al., 2018). Currently there is no cure for Rett syndrome and palliative care directed toward ameliorating a patient’s specific symptoms is the only means to help these individuals. Previous studies have demonstrated that RTT is not neurodegenerative and that reactivation of *MECP2* expression can reverse phenotypes in a mouse model of Rett Syndrome (Guy J. et al., 2007). This earlier work on RTT generated an inducible mouse model by insertion of a Lox-Stop cassette, under control of its own promoter and regulatory elements allowing for the conditional expression of *MECP2*. These mice survive for ~10 weeks in males and display striking neurological symptoms in females by 12–16 weeks. Re-expression of *MECP2* by ~80% resulted in a near loss of advanced neurological symptoms in mature adult animals, extending survival in males past 30 weeks and reducing neurological severity by 50% in females (Guy J. et al., 2007). Based on these results, innovative therapies capable of delivering functional MeCP2 to affected cells need to be developed.

An autologous hematopoietic stem cell gene therapy (HSCGT) approach to treat Rett Syndrome has the potential to offer a treatment to affected patients. This would be accomplished through a process called cross-correction where gene modified cells of the hematopoietic lineage provide constitutive and systemic expression of functional MeCP2. Human CD34+ hematopoietic stem and progenitor cells (HSPC) transduced with a lentiviral vector expressing *MECP2* can engraft in the bone marrow of transplanted patients and their immune cell progeny would deliver functional MeCP2 throughout the body including the central nervous system (CNS). Specifically, myeloid cells which migrate to the brain and reside as microglia would have the ability to deliver MeCP2 to affected neurons (Capotondo A. et al., 2012; Sergijenko A. et al., 2013).

Recent work with other monogenic disorders including those directly affecting neurons has demonstrated the success of this cross-correction approach. These include cerebral adrenoleukodystrophy (CALD), metachromatic leukodystrophy (MLD), Tay-Sachs/Sandhoff disease, Angelman syndrome (AS), SYNGAP1, and mucopolysaccharidoses (MPS) (Biffi A. et al., 2013; Beegle J. et al., 2020; Adhikari A. et al., 2021; Tucci F. et al., 2021; Eichler F. et al., 2017; Larkin, 2022; Fraldi A. et al., 2018; Penati R. et al., 2017; Anderson JS. et al., 2024). In our experiments with AS and SYNGP1, significant rescue in disease-specific phenotypes were observed using immunodeficient mouse models engrafted with human CD34+ HSPC transduced with lentiviral vectors expressing modified forms of Ube3a or Syngap1 (Adhikari A. et al., 2021; Anderson JS. et al., 2024). The results observed in these studies lead us to believe that this strategy can benefit other genetic neurodevelopmental disorders (NDDs) including Rett syndrome. This is the main hypothesis behind our current experiments described here. If HSPC transduced with an *MECP2* expressing lentiviral vector can engraft and deliver functional MeCP2 to affected cells, reversal and/or prevention of disease progression could occur. Several advantages of this strategy include: 1) autologous HSPC are derived from each patient and thus less likely to cause complicating immune responses that require life-long immune suppression, 2) genetically engineered HSPC-derived cells overexpressing a modified form of MeCP2 are capable of secreting functional enzyme in therapeutic quantities, 3) genetically engineered HSPC-derived cells infiltrate the CNS and potentially all tissues in the body where they work as enzyme donors thus working as a systemic therapy, 4) lentiviral vector-mediated modifications can lead to constitutive long-term expression of MeCP2 from HSPC-derived cells and may allow for a one-time treatment, and 5) neurons are not transduced directly, minimizing the potential for vector-mediated neurotoxicity.

In our current study, we evaluated whether an HSCGT approach could be effective in treating Rett syndrome phenotypes. After transplantation of human CD34+ HSPC transduced with a lentiviral vector expressing a modified form of MeCP2 into an immunodeficient mouse model of Rett syndrome, improvement of disease-related phenotypes was observed. These proof-of-concept results demonstrate that this treatment strategy has the potential to provide long-term therapeutic benefit for patients with Rett syndrome. It also supports the possible use of this approach for the treatment of other genetic NDDs.

## 2 Results

### 2.1 Functionality of the *MECP2* vector and expression in transduced B lymphocytes

**FIGURE 1:**
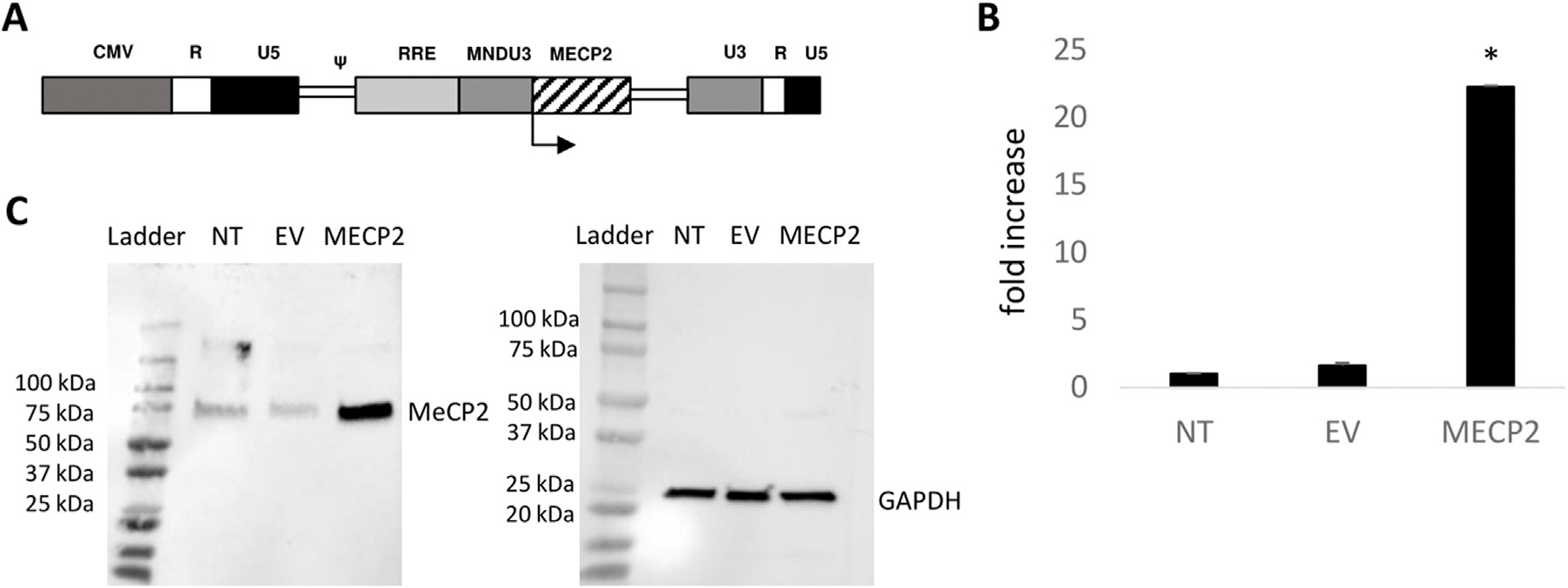
Functionality of an MECP2 expressing lentiviral vector: **(A)** The modified *MECP2* gene was cloned into a third-generation self-inactivating lentiviral vector. The control empty vector (EV) consists of the same vector backbone without the *MECP2* transgene. **(B)** Rett syndrome-affected B lymphocytes were left nontransduced (NT) or transduced with the EV or *MECP2* vector. Total RNA was extracted and *MECP2* transcripts were quantified by QPCR (N = 3). Data is presented as a fold-increase in expression normalized to 1.0 from the NT data. Mean and standard error of the mean are shown. **(C)** Total protein was extracted from the cells and evaluated for expression of MeCP2 (~75 kDa) by Western blot. GAPDH (~25 kDa) was used as an internal loading control. *p < 0.001.

*MECP2* lentiviral vectors were successfully generated and we obtained average titers of 1.80 × 10^9^ TU/mL. To evaluate the levels of expression of MeCP2 from the lentiviral vector in transduced cells, human B lymphocytes affected by Rett Syndrome and purchased from a commercial source were transduced with the *MECP2* lentiviral vector. As displayed in Figure 1B, we observed >22-fold expression of *MECP2* RNA, in *MECP2* vector transduced cells compared to nontransduced (NT) (p < 0.001) and empty vector (EV) (p < 0.001) control cells, as measured by QPCR. All values were normalized to internal control GAPDH levels prior to comparison. As displayed in Figure 1C, corresponding overexpression of MeCP2 protein (~75 kDa) was observed in cells transduced with the *MECP2* lentiviral vector compared to NT and EV control cells, as detected by Western blot. GAPDH (~25 kDa) was used as an internal loading control. These results demonstrated the functionality of the lentiviral vector with expression of *MECP2*/MeCP2 in transduced Rett-syndrome affected B cells.

### 2.2 CFU assay and derivation of macrophages

**FIGURE 2:**
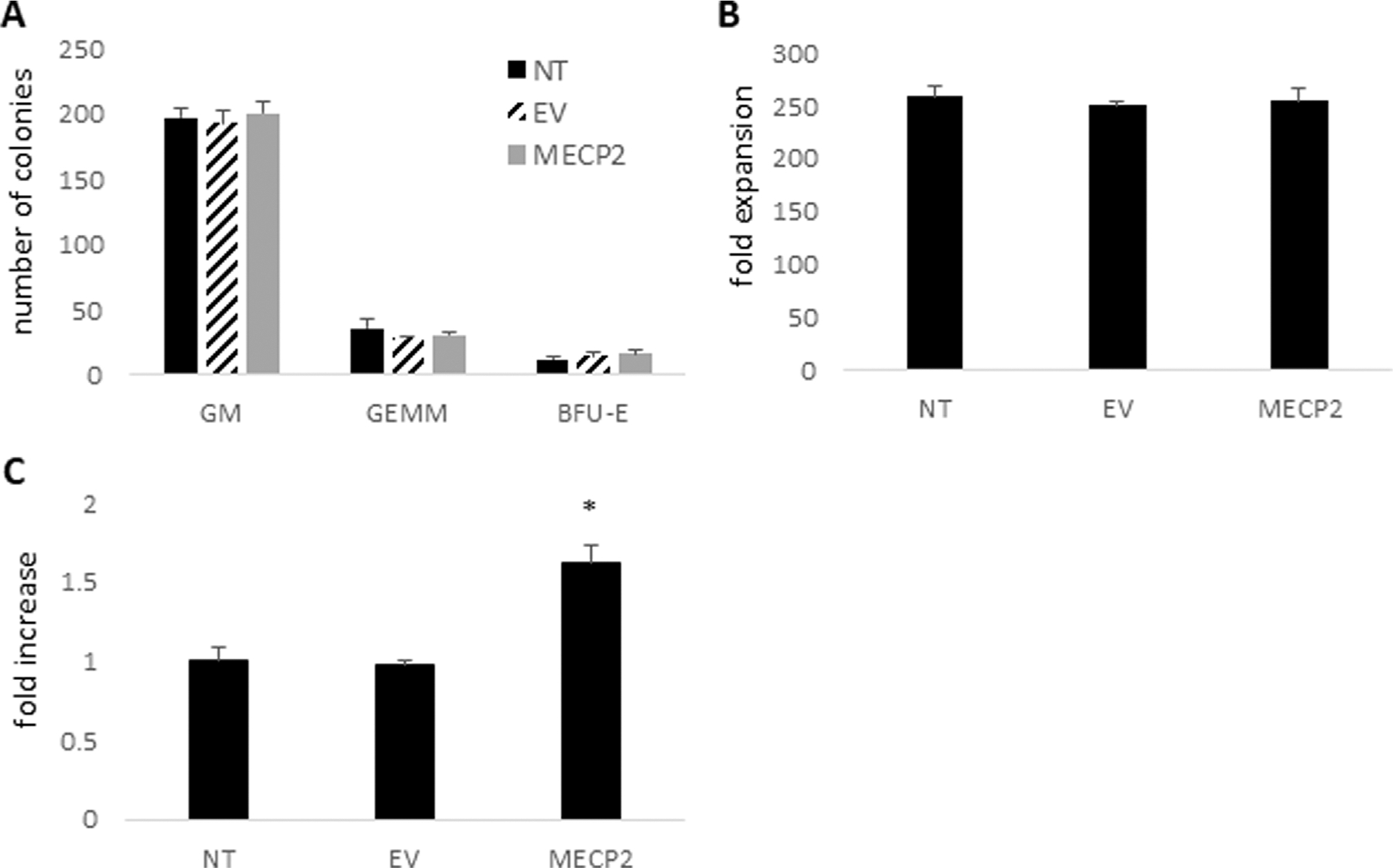
CFU assay, cell expansion, and expression of MECP2 in human CD34+ derived macrophages: Human CD34+ HSPC were left nontransduced (NT) or transduced with the empty vector (EV) or the *MECP2* vector and cultured in a CFU assay with methylcellulose media. **(A)** Total GM, GEMM, and BFU-E colonies were counted. **(B)** Total cell expansion was evaluated by comparing the number of input cells to the number of cells after the CFU assay. **(C)** Total RNA was extracted from the CD34+ derived macrophages post-CFU assay and *MECP2* transcripts were quantified by QPCR. Data is presented as a fold-increase in expression normalized to 1.0 from the NT data. Mean and standard error of the mean are shown. *p < 0.01.

Due to the introduction and overexpression of the modified *MECP2* gene in human CD34+ HSPC upon transduction with the *MECP2* lentiviral vector, there is a possibility of dysfunctional differentiation of the gene modified human CD34+ HSPC. Therefore, to evaluate the differentiation capacity of the cells to develop into various cell lineages, an *in vitro* CFU assay was performed. This assay is also a required evaluation by the FDA for calculating vector copy numbers per transduced cell and when evaluating patient drug products in clinical trials. Therefore, we included the CFU assay in this study to initially evaluate the safety of the *MECP2* vector transduced cells. As displayed in Figure 2A, similar numbers, on average, of GM, GEMM, and BFU-E colonies were observed in cultures from the *MECP2* (GM = 201, GEMM = 31, BFU-E = 16) vector transduced human HSPC compared to the control NT (GM = 197, GEMM = 36, BFU-E = 12) and EV (GM = 193, GEMM = 28, BFU-E = 15) cultures. As displayed in Figure 2B, similar total expansion of the *MECP2* vector transduced cells (255-fold) was also observed compared to the control NT (259-fold) and EV (251-fold) cells.

Following the CFU assay, the cells were further differentiated into macrophages. Flow cytometry was performed to evaluate levels of normal macrophage cell surface markers. The cells transduced with the *MECP2* lentiviral vector displayed similar levels of CD14 (90.3%), CD4 (93.4%), and HLADR (59.6%) compared to NT cells (96.6% CD14, 91.7% CD4, and 55.8% HLADR) and EV cells (96.3% CD14, 92.1% CD4, and 51.2% HLADR) As displayed in Figure 2C, significant overexpression (>1.6-fold) of *MECP2* RNA was also detected in the *MECP2* vector transduced macrophages compared to the control NT (p < 0.01) and EV (p < 0.01) cells.

These results demonstrated that normal expansion and differentiation into the myeloid lineage cells could be achieved after transduction of human CD34+ HSPC with the *MECP2* expressing lentiviral vector.

### 2.3 Improvement of locomotor phenotypes in MECP2 vector treated BRM mice

To evaluate whether human CD34+ HSPC transduced with an *MECP2* lentiviral vector could improve phenotypes associated with Rett syndrome, cells were transplanted into female immunodeficient BRM mice (B6-*Rag2*^−/−^*Mecp2*^−/+^). We utilized 2–5 day old newborn and 8 week old adult mice for these experiments. Sixteen weeks post-transplant, these mice were subjected to rotarod and open field assays to assess motor abilities. Heterozygous *Mecp2*^−/+^ mice exhibit decreased latency times to fall from the rotarod due to decreased clasping abilities, as well as decreased movements in an open field assay due to increased bodyweights and mobility issues (Guy J. et al., 2001; Samaco RC. et al., 2013).

**FIGURE 3:**
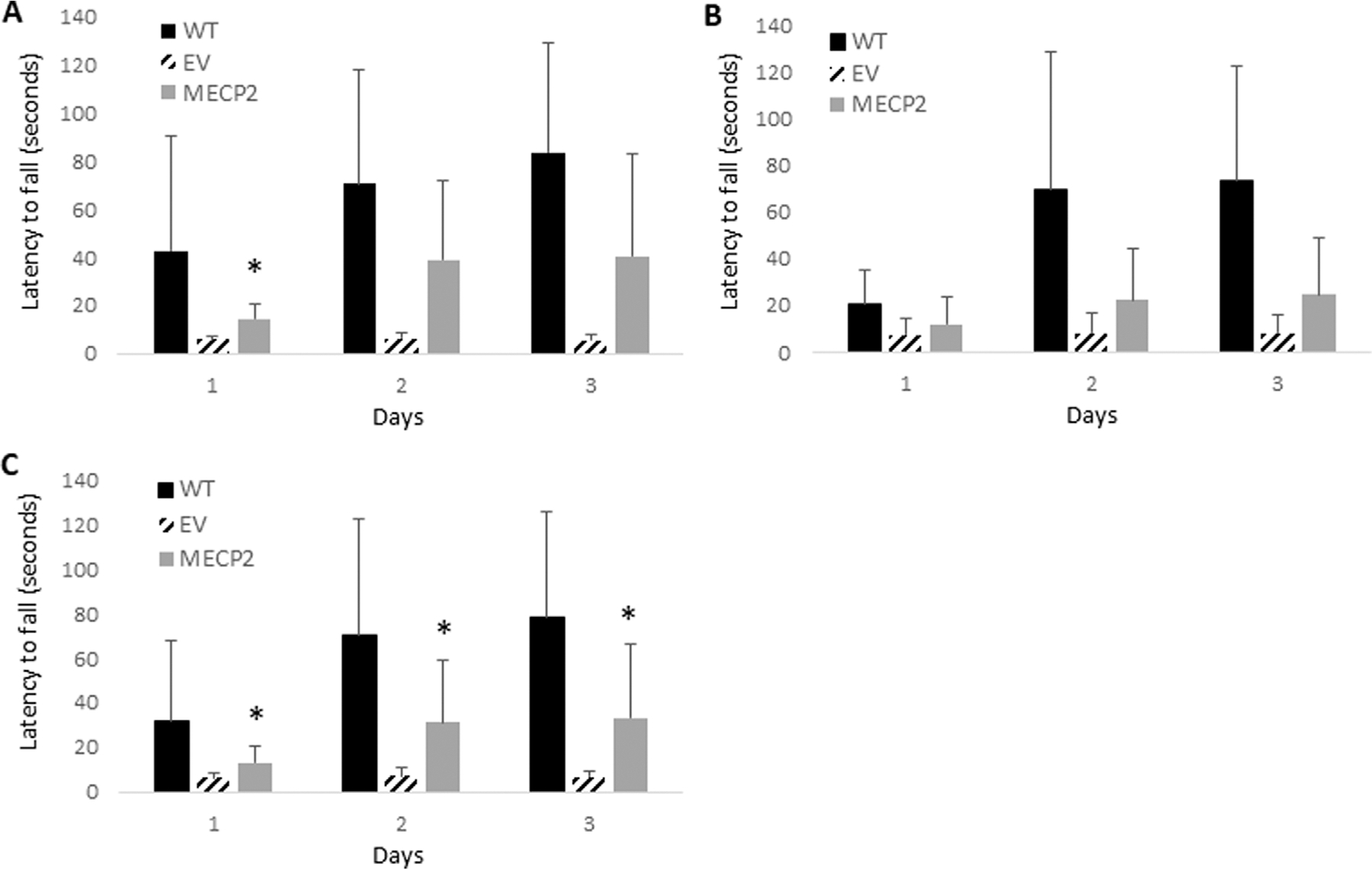
Rotarod analysis of BRM mice transplanted with MECP2 vector transduced human CD34+ HSPC: BRM mice were transplanted with human CD34+ HSPC transduced with either the empty vector (EV) or the *MECP2* vector *MECP2*). Nontransplanted *MECP2* wild type (WT) (B6-*Rag2*^−/−^ *Mecp2*^+/+^) mice were used as controls. Sixteen weeks post-transplant, the mice were subjected to a rotarod assay for three consecutive days. Total latency time to fall was measured with a maximum time of 5 min. Data displayed from transplanted **(A)** newborn mice (N = 4), **(B)** adult mice (N = 4), or **(C)** a combination of all mice (N = 8). Mean and standard error of the mean are shown. *p < 0.05.

As displayed in Figure 3A, improvement in latency time to fall in a rotarod assay was observed in the *MECP2* vector transplanted (N = 4) newborn mice on all three testing days as compared to the EV group (N = 4). A significant level of improvement was observed on day 1 (p = 0.027). The *MECP2* vector transplanted newborn mice also demonstrated the ability to learn and adapt to the rotarod during the three consecutive days of the assay, as the latency time to fall increased from days 1–3 from 14.4 s to 40.4 s as compared to the EV group, which did not demonstrate an increase from days 1–3 (5.8 s–5.3 s). As displayed in Figure 3B, increased improvement in latency time to fall was demonstrated in the *MECP2* vector group (N = 4) transplanted as adult mice on all three testing days compared to the EV group (N = 4), however without significance (day 1 p = 0.39, day 2 p = 0.22, and day 3 p = 0.09). The *MECP2* vector transplanted adult mice also demonstrated the ability to learn during the three consecutive days of rotarod testing as the latency time to fall increased from days 1–3 from 12.0 s on day 1–24.5 s on day 3.

Due to low numbers of transplanted mice in each of the cohorts grouped by age of transplantation, the data from the newborn and adult mice were combined and re-analyzed. This can be done when there is no significant difference between the control groups with which the comparison is being performed. When comparing the EV group transplanted as newborns versus adults, no statistically significant difference in performance was observed on days 1 (p = 0.40), 2 (p = 0.63), or 3 (p = 0.33). When comparing the WT group used for the newborn versus adult experiments, no statistically significant difference in performance was observed on days 1 (p = 0.20), 2 (p = 0.98), or 3 (p = 0.66). As displayed in Figure 3C, significant improvement in latency time to fall was observed in *MECP2* transduced cell transplanted BRM mice (N = 8) on all 3 days as compared to the EV control group (N = 8) (day 1 p = 0.025, day 2 p = 0.028, and day 3 p = 0.037). However, we did not observe a full reversion of phenotypes in the *MECP2* transplanted mice to WT levels as the difference between the *MECP2* and WT groups were still significantly different by day 3 (p = 0.015). When grouped together, the ability to learn during the three consecutive days of the assay was observed as the latency time to fall increased from days 1–3 from 13.3 s to 33.3 s in the *MECP2* cell transplanted mice.

**FIGURE 4:**
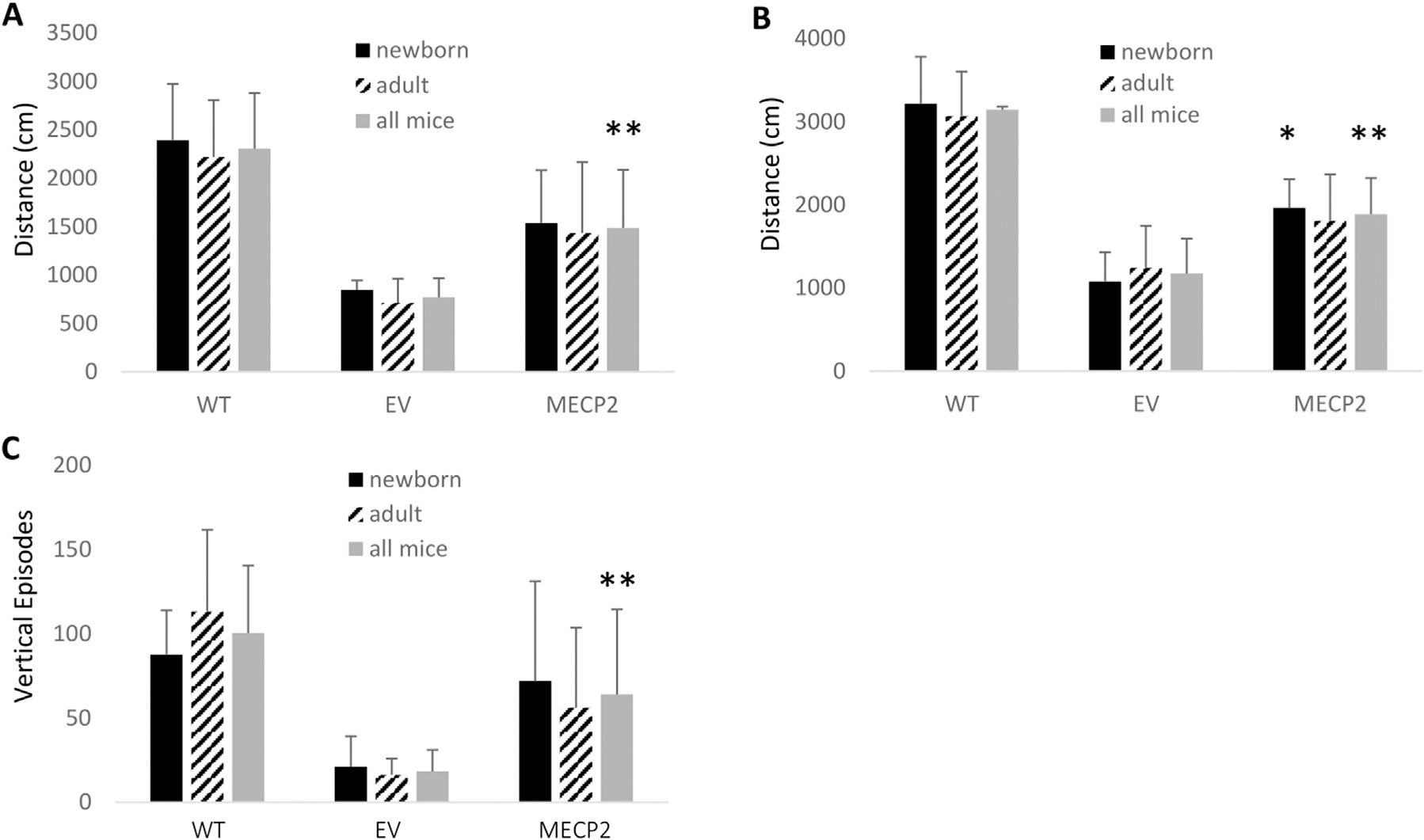
Open field analysis of BRM mice transplanted with MECP2 vector transduced human CD34+ HSPC: BRM mice were transplanted with human CD34+ HSPC transduced with either the empty vector (EV) or the *MECP2* vector (*MECP2*). Nontransplanted *MECP2* wild type (WT) (B6-Rag2^−/−^ Mecp2^+/+^) mice were used as controls. Sixteen weeks post-transplant, the mice were subjected to a 15-minute open field assay to evaluate **(A)** total distance traveled, **(B)** total horizontal activity, and **(C)** vertical rearing activity. Data is displayed from transplanted newborn mice (N = 4), adult mice (N = 4), or a combination of all mice (N = 8). Mean and standard error of the mean are shown. *p < 0.05 and **p < 0.01.

To further evaluate motor abilities of mice transplanted with the *MECP2* vector transduced cells, an open field assay was conducted. Again, data were grouped and analyzed for newborn transplanted alone (N = 4), adult transplanted alone (N = 4 per group), and as combined groups (N = 8). As displayed in Figure 4A, improvement in total distance traveled was observed in the *MECP2* vector group compared to the EV control group regardless of the age the mice were transplanted. However, when both *MECP2* vector mouse groups were combined to improve statistical power and then compared to the EV group, a statistically significant (p = 0.01) improvement was observed. As displayed in Figure 4B, improvement in horizontal activity was also observed in the *MECP2* vector group compared to the control EV group regardless of the age the mice were transplanted. However, a significant increase was observed in the *MECP2* newborn transplanted group when analyzed by itself as well as when combined with adult transplanted *MECP2* vector mice, when compared to the EV group (p = 0.02 and p = 0.007), respectively). Furthermore, significant improvement in vertical rearing activity was observed in the *MECP2* vector group compared to the control EV group regardless of the age the mice were transplanted, as displayed in Figure 4C. When all mice were combined and reanalyzed, a significant (p = 0.037) improvement in vertical rearing activity was observed compared to the EV group. Reversion to WT activity levels for total distance and horizontal activity was not observed in the combined *MECP2* vector group as the differences were still significant (p < 0.05). However, there was not a significant difference (p = 0.06) between WT and the combined *MECP2* group when analyzing vertical rearing activity.

At the conclusion of these studies, the peripheral blood of the mice was collected and analyzed by QPCR for vector copy number to evaluate the levels of the gene modified cell engraftment. EV cell transplanted mice displayed engraftment levels between 0.39 and 1.78 average vector copies per cell in the peripheral blood. *MECP2* vector transduced cell transplanted mice displayed engraftment levels between 0.78 and 1.68 average vector copies per cell in the peripheral blood. This demonstrated that the *MECP2* vector transduced cells were capable of maintaining engraftment from the time of transplantation through the behavioral and motor experiments. Collectively, these results demonstrate that Rett syndrome-related motor phenotypes could be improved after transplantation of human HSPC transduced with an *MECP2* lentiviral vector.

### 2.4 Increased MeCP2 expression in the brain tissue of MECP2 vector treated BRM mice

**FIGURE 5:**
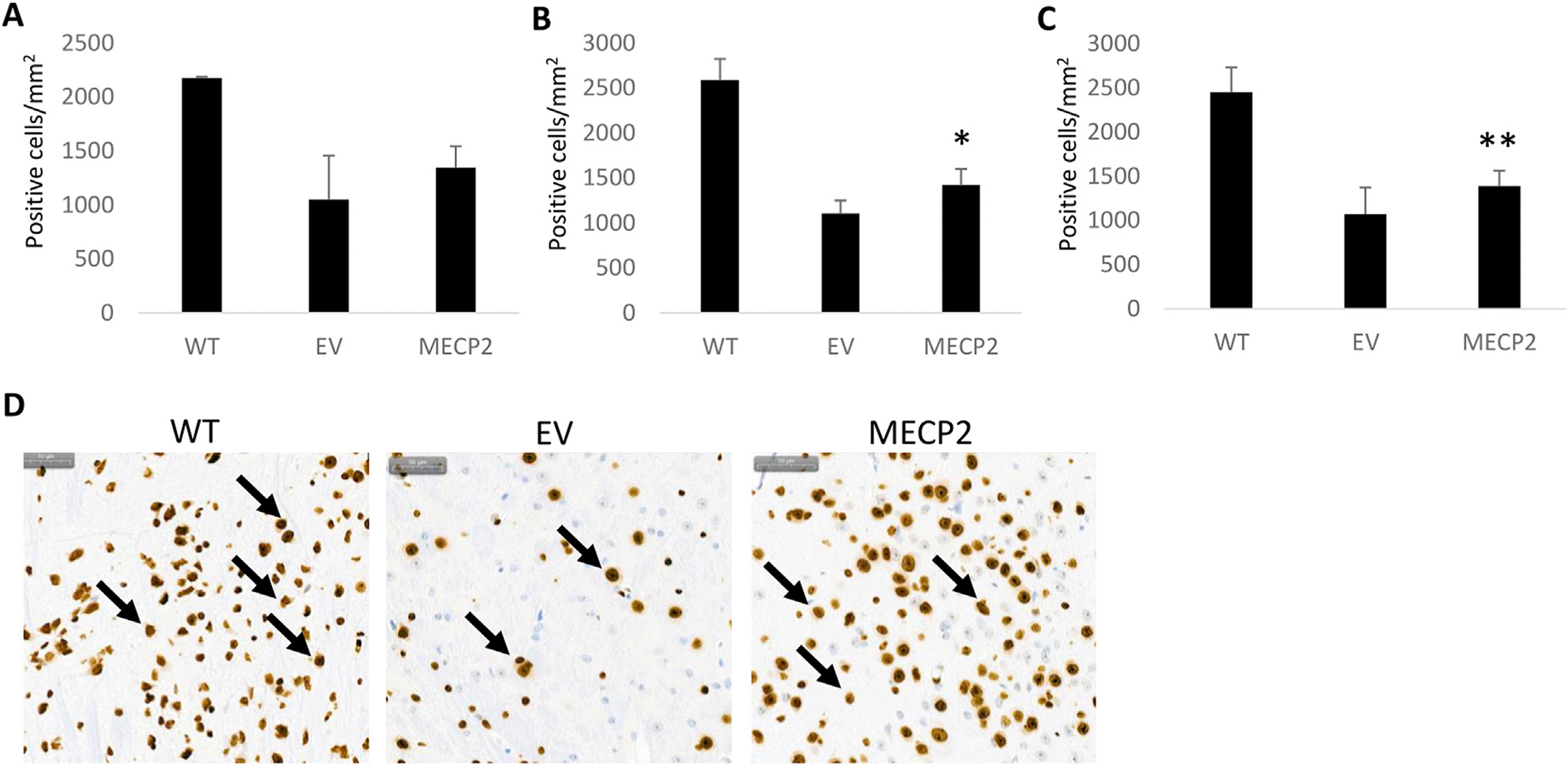
Immunohistochemical detection of MECP2 in brain tissue obtained from BRM mice: Sagittal sections of brain tissue obtained from the nontransplanted wild type (WT), empty vector (EV) transduced, or *MECP2* expressing (*MECP2*) vector transduced cell transplanted mice were labeled with a monoclonal antibody for mouse *MECP2* (N = 7). Enumeration of positive cells was performed and compared between the groups. Data displayed from transplanted **(A)** newborn mice, **(B)** adult mice, or **(C)** a combination of all mice. **(D)** Representative stained images from the brain tissues are displayed for the WT, EV, and MECP2 groups with arrows indicating positive cells. The scale bar in the upper left represents 50 μm. Mean and standard error of the mean are shown. *p = 0.05 **p = 0.03.

To determine whether an increased level of MeCP2 was present in the mice transplanted with the *MECP2* vector transduced cells, immunohistochemistry was performed on brain tissue. As displayed in Figure 5, an increase in expression of MeCP2 was detected in the brains of newborn and adult mice transplanted with the *MECP2* vector transduced cells as compared to EV mice. A significant increase in expression was observed in the adult transplanted *MECP2* group (p = 0.05) (Figure 5B) and when both adult and newborn transplanted *MECP2* groups were combined (p = 0.03) (Figure 5C). Reversion to WT levels of MeCP2 expression was not observed as the values were still significantly different (p < 0.05). Representative images from the stained tissues are displayed in Figure 5D with arrows indicating MeCP2 positive cells. These results demonstrate that transplantation of *MECP2* vector transduced human CD34+ HSPC can lead to an increased level of MeCP2 in the brain.

## 3 Discussion

Currently, there are no effective treatments for Rett syndrome and palliative care is the only option for affected patients. Previously, gene and epigenetic therapeutics targeted to RTT used viral reintroduction of *MECP2* (Gadalla KK. et al., 2013; Sinnett and Gray, 2017; Sinnett SE. et al., 2017; Alvarez-Saavedra M. et al., 2007; Qian J. et al., 2023). Re-expression of MeCP2 resulted in the loss of the majority of neurological symptoms in adult animals, extending the survival time of males past 30 weeks and reducing neurological severity in females. Key aspects of this work are corroborated in our results, such as the adult tailored reinstatement of *MECP2*. This report extends upon the previously relied upon “neurological severity score” which is subjective and categorical, using continuous variables indices. Our stem cell gene therapy approach which allows for the long-term systemic delivery of functional MeCP2 may offer a potential way to reverse and/or halt disease progression before it begins.

Although early in development, our current proof-of-concept experiments, we evaluated the efficacy of*MECP2* expressing lentiviral vector transduced human CD34+ HSPC transplanted in an immunodeficient mouse model of Rett syndrome. This strategy combines the benefit of long-term engraftment of hematopoietic stem and progenitor cells and permanent integration of lentiviral vectors to generate a potential one-time, life-long treatment for the patient. Further, it does not rely on the correction of each individually affected cell as the transplanted gene modified cells constitutively express and deliver therapeutic proteins throughout the body. This therapeutic strategy relies on the engraftment of gene modified CD34+ HSPC and their subsequent immune cell progeny to circulate systemically and migrate to most sites in the body, including myeloid progenitors into the CNS where they reside as microglia (Capotondo A. et al., 2012; Sergijenko A. et al., 2013). These gene modified microglia would then express MeCP2 and deliver it to affected neurons through a process called cross-correction. This strategy has been effective both preclinically and clinically in other monogenetic diseases including CALD, MLD, MPS, AS, SYNGAP1, and Tay-Sachs/Sandhoff disease. The success of this approach for these other disorders relies on the natural mechanism of the respective disease-related proteins to be secreted from cells and taken up by nearby cells through the mannose-6-phosphate receptor. However, as MeCP2 is strictly found intracellular, mainly in the nucleus, it was not an obvious therapeutic strategy to use for Rett syndrome. Based on our previous success in other neurodevelopmental disorders including AS and SYNGAP1 using modified forms of their respective therapeutic proteins, we employed a similar strategy when developing the therapeutic *MECP2* expressing lentiviral vector (Adhikari A. et al., 2021; Anderson JS. et al., 2024). Modifications were made to the *MECP2* gene sequence to allow secretion of MeCP2 from the gene modified cells after transplantation and uptake by nearby cells. However, since MeCP2 is a nuclear protein, it might be more difficult to deliver therapeutically relevant levels of the protein as our strategy relies on other cells to deliver functional MeCP2 to affected cells.

Due to the modifications made to the *MECP2* transgene, we needed to first evaluate the functionality of the vector. Both *MECP2* transcripts and MeCP2 protein were successfully detected in cells transduced with the *MECP2* lentiviral vector *in vitro*. We also observed normal cell expansion, differentiation into CFU colonies, and differentiation to mature macrophages after transduction. To evaluate the *in vivo* efficacy of the *MECP2* lentiviral vector, we created an immunodeficient mouse model of Rett syndrome which allowed us to transplant human CD34+ HSPC without the concern for rejection due to the introduction of the *Rag2*^−/−^ knockout. This also allowed us to evaluate the efficacy of human CD34+ cells which would be used in a clinical setting. We chose the *Rag2*^−/−^ knockout as the genotype to create the immunodeficiency based on previous work demonstrating arrested T and B cell development at the pro-cell stage, thereby creating a non-leaky phenotype (Hao and Rajewsky, 2001). Upon crossing the *Rag2*^−/−^ model with the *Mecp2*^−/+^ model, the introduction of the Rag2-null genotype did not alter or confound any of the Rett syndrome-related phenotypes in the original MeCP2-deficient model. These mice retained the typical Rett mouse model phenotypes, including weight gain, decreased latency to fall from the rotarod, and mobility issues in an open field assay.

In an attempt to develop a stem cell gene therapy for Rett syndrome, we transplanted *MECP2* vector transduced human CD34+ cells into the BRM model and evaluated motor skills in rotarod and open field activities. Only heterozygous *Mecp2*^−/+^ females were evaluated since homozygous females (*Mecp2*^−/−^) and hemizygous males (*Mecp2*^−/*y*^) die within 10 weeks post-birth, limiting engraftment potential. In our initial experiments, we also evaluated this therapeutic approach in 2–5 day old newborn male hemizygous BRM mice, however, no significant extension of lifespan was observed (data not included). The early death of *MECP2* knockout mice is a limitation of this model as it only allows for therapeutic evaluation of heterozygous females.

When utilizing female heterozygous mice in the *MECP2* group, an increase in latency time to fall in the rotarod assay and increased mobility measured in the open field assay were observed compared to the EV control group. Our results from the rotarod assay also demonstrated the *MECP2* group’s ability to learn as the increase in latency time to fall was measured over the course of the three-day testing period. When evaluating efficacy, the *MECP2* group was directly compared to the EV control group as these mice received vector transduced cells and busulfan conditioning, with the only difference being the lack of the therapeutic *MECP2* transgene in EV transduced cells.

When designing the experiments to measure efficacy, we also wanted to evaluate whether there was a therapeutic window of treatment. By transplanting the mice as either 2–5-day old newborns or 8-week old adults, we evaluated whether age of transplantation demonstrated any difference in phenotypic rescue. We observed a significant change in motor abilities when analyzing the *MECP2* group transplanted as newborns on day 1 of the rotarod assay and with total horizontal activity in the open field assay. No significant differences were observed in any of the data with the adult transplanted *MECP2* subgroup. However, there was a trend toward WT with all data collected in the *MECP2* groups.

To increase statistical power, we combined and re-analyzed the data from the newborn and adult mice into their specific groups, either WT, EV, or *MECP2*. This can be performed when there is no statistical significance between the values measured in the EV newborn and adult transplanted mice and between the values in the WT newborn and adult mice, which aligns with our observations. After combining data from all mice transplanted with the *MECP2* vector transduced cells, a significant difference was observed on all days of the rotarod assay and with all parameters in the open field assay in comparison to the EV group. While we observed *in vivo* efficacy of motor function correction which is one of the most dramatic phenotypes of Rett syndrome, other clinical phenotypes including cognition and sleep were not evaluated.

To further investigate the improvement in motor abilities after *MECP2* vector transduced cell transplantation, we performed IHC on brain tissues to measure MeCP2 expression. We observed a significant increase in MeCP2 expression in the tissues of the *MECP2* vector transduced cell transplanted mice compared to the EV group. Since the mice in the EV control group are heterozygous for *Mecp2*^−/+^ rather than full knockouts, expression was still detected in these samples. A limitation of this analysis includes a lack of observing the exact percent or fold increase in MeCP2 in the brain tissues since IHC is semi-quantitative. Future work including either Western blots or ELISAs would improve these results. Also, if *MECP2*^−/−^ knockout mice were viable for transplantation, it is possible we would have observed an even greater difference in MeCP2 expression. The detection of the gene modified cells post-analysis demonstrated that the *MECP2* vector transduced cells were capable of maintaining engraftment from the time of transplantation through the behavioral and motor experiments. Further long-term engraftment studies of the *MECP2* vector transduced cells will add to the ability of this strategy transplating to a potential one-time treatment.

Our group has expanded the use of the cross-correction strategy to not only include lysosomal storage diseases and leukodystrophies but also NDDs as demonstrated with our work on AS and SYNGAP1. However, the exact mechanism of how this approach works for Rett syndrome, considering that MeCP2 is an intracellular and nuclear localized protein, requires additional investigation beyond the concept of our current work. Future work will help to identify specifically which human cells express and deliver MeCP2 to neurons and in what concentration. Subsequent experiments will also help optimize the degree of MeCP2 expression to provide greater improvement of Rett syndrome related phenotypes toward wild type levels. These analyses are critical as it was previously shown that duplications of the *MECP2* gene will impair CNS function leading to duplication syndrome (Lombardi et al., 2015).

Based on our data, we report a potential novel stem cell gene therapy approach for treatment of Rett syndrome. These results not only provide the first step to a novel therapeutic approach aimed at disease treatment rather than palliative care for Rett syndrome patients, but they also provide the groundwork for the development of stem cell gene therapies for other NDDs.

## 4 Methods

### 4.1 MECP2 lentiviral vector

A third-generation lentiviral vector was used to develop the *MECP2* expressing vector (named *MECP2*). A modified version of the mouse *MECP2* gene was synthesized, inserted downstream and under the control of a constitutive MNDU3 promoter within the vector backbone (Figure 1A). Sequence verification was then performed (Laragen, Culver City, CA, United States). The empty vector backbone (named EV) was used in the experiments as a control for vector transductions.

The *MECP2* lentiviral vectors were generated in human embryonic kidney (HEK)-293 cells by transient transfection using polyethyleneimine with a 1:1:5:5 ratio of: a) vesicular stomatitis virus glycoprotein (VSVG) envelope plasmid, b) a Rev gene expression plasmid, c) a packaging plasmid containing the capsid and reverse transcriptase genes, d) and either one of the transfer plasmids, the control EV or the *MECP2* expression plasmids. After overnight transfection, the media was changed to serum free XVIVO-10. After waiting for 48 h, the vector supernatants were collected and concentrated by ultrafiltration. Lentiviral vector titers were then measured by transduction of HEK-293 cells followed by total DNA extraction (Promega, Madison, WI, United States) and evaluation of vector copies by quantitative PCR (QPCR). The vector specific primer and probe sequences used for vector titering are as follows: forward primer: 5′-ACCTGAAAGCGAAAGGGAAAC-3′ reverse primer: 5′-CGCACCCATCTCTCTCCTTCT-3′ probe: 5′-AGCTCTCTCGACGCAGGACTCGGC-3’. QPCRs were performed using a StepOne Real Time PCR System (ThermoFisher, Carlsbad, CA, United States).

### 4.2 MECP2-deficient B lymphocytes and human CD34+ HSPC

B lymphocytes deficient in *MECP2* expression were purchased from a commercial source (Coriell, Camden, NJ, United States). These cells were cultured in RPMI media (ThermoFisher, Carlsbad, CA, United States) with the addition of 15% fetal bovine serum and 5% L-glutamine. For the experiments described below, the B lymphocytes were left either nontransduced (NT), transduced with the EV vector control (EV), or transduced with the *MECP2* vector (*MECP2*) at a multiplicity of infection (MOI) of 5. Protamine sulfate (10 mg/mL) was added and the transductions were performed for a minimum of 3 h at 37°C.

Human CD34+ HSPC were purchased from a commercial source (Stem Cell Technologies, Vancouver, BC, Canada). These cells were cultured for 18 h in XVIVO-10 media (Lonza, Anaheim, CA, United States) with the addition of 100 ng/mL Flt-3 ligand, thrombopoietin (TPO), and stem cell factor (SCF) (R&D Systems, Minneapolis, MN). After culturing, the CD34+ cells were left either NT, transduced with the EV vector, or transduced with the *MECP2* vector at an MOI of 5. Protamine sulfate (10 mg/mL) and Lentiboost (1:100 dilution) (Sirion Biotech, Cambridge, MA, United States) were added. Transductions were left for a minimum of 3 h at 37°C.

### 4.3 Colony forming unit (CFU) assays

Human CD34+ HSPC were left as either NTor transduced with the EV vector or the *MECP2* vector as described above. Post-transduction, 500 total cells from each group were pipetted into cytokine-enriched MethoCult medium (Stemcell Technologies, Vancouver, BC, Canada), gently vortexed, plated into 35 mm diameter culture dishes, and cultured for 14 days. After this time period, total granulocyte/macrophage (GM), granulocyte/erythrocyte/megakaryocyte/macrophage (GEMM), and burst forming unit-erythroid (BFU-E) colonies were counted by microscope visualization. Fold expansion of total cells was also calculated by counting the numbers of output cells on a hemacytometer. Experiments were performed in triplicate.

### 4.4 *In vitro* derivation of macrophages and their phenotypic profile

After completion of the CFU assays, the cells were removed from the methylcellulose media by the addition of complete DMEM supplemented with 10% FBS. The cells were spun down by centrifugation and further cultured in DMEM supplemented with 10% FBS, 10 ng/mL of granulocyte/macrophage colony stimulating factor (GM-CSF), and 10 ng/mL macrophage colony stimulating factor (M-CSF) (R&D Systems, Minneapolis, MN, United States) for 4 days to derive mature macrophages. Once the macrophages developed, these cells were analyzed by flow cytometry for surface expression of myeloid-specific cell surface markers, CD14, CD4, and HLA-DR. The macrophages were lifted from the culture dishes and stained with 10 μl of either a phycoerythrin (PE)-conjugated CD14 (catalog# 555398) antibody, a PE-conjugated CD4 (catalog# 555347) antibody, or a PE-conjugated HLADR antibody (catalog# 555561) (BD Biosciences, San Jose, CA, United States). Flow cytometry was then performed on a Becton Dickinson LSRII flow cytometer.

### 4.5 Quantitative PCR (QPCR)

To detect *MECP2* mRNA expression upon transduction with the *MECP2* lentiviral vector, QPCR was performed. Cells, wither the cultured B lymphocytes affected by Rett syndrome or the macrophages derived from the vector transduced human CD34+ HSPC were collected from their culture plates and total RNA was extracted using RNA STAT-60 (amsbio, Cambridge, MA, United States) according to the manufacturer’s protocol. Briefly, the cells were centrifuged down and resuspended in PBS. The RNA STAT-60 reagent (1 mL) was added to the resuspended cells and allowed to sit for 5 min. Chloroform (100 μl) was then added and the suspension was vortexed for 10 s followed by a resting period of 5 min. The suspension was then centrifuged to allow for the phase separation. The clear aqueous phase containing the RNA was then collected and put into a clean centrifuge tube. Seventy percent ethanol was added to the aqueous phase, mixed gently, and centrifuged to concentrate the RNA. The supernatant was then discarded and the RNA pellets were resuspended in RNase-free water. Synthesis of subsequent cDNA was then performed using a Taqman Reverse Transcription Reagents kit (ThermoFisher, Carlsbad, CA, United States) according to the manufacturer’s protocol. QPCR was then performed on the synthesized cDNA using a mouse *MECP2*-specific primer and probe set: forward primer: 5′-CCTCCTTGGACCCTA ATGATT-3′, reverse primer: 5′-CCTGGAGCTTTGGGAGATTT-3′, probe: 5′-AGAGCAGAAACCACTAAGAAGCCC-3′ using a Taqman Universal PCR Master Mix kit (ThermoFisher, Carlsbad, CA, United States) according to the manufacturer’s protocol. QPCR experiments were performed in triplicate.

### 4.6 Western blot

To perform the Western blots, total protein from the Rett syndrome-affected B lymphocytes either NT or transduced with the EV or *MECP2* vectors was performed by multiple freeze thaw cycles alternating from freezing on dry ice to thawing in a 37°C water bath (six total freeze-thaw cycles). The suspension was then centrifuged to remove cell debris and the supernatant was collected followed by the addition of a protease inhibitor. Protein quantities were then calculated by Bradford assay. Thirty micrograms total protein from each group and the Precision Plus Protein Dual Color Ladder (BioRad, Hercules, CA, United States) were separated on a 4%–20% Mini-PROTEAN TGX gel (BioRad, Hercules, CA, United States) with Tris-Glycine SDS Running Buffer (Invitrogen, Carlsbad, CA, United States). Protein transfers were then performed onto a nitrocellulose membrane. This membrane was then incubated in blocking buffer which consisted of 5% bovine serum albumin in TBST [Tris-buffered saline with Tween-20 (50 mm Tris, 150 mm NaCl, 0.1% Tween-20)] for 30 min at room temperature. The membranes were then incubated with primary rabbit anti-MeCP2 polyclonal antibody (catalog# PA1–888) (ThermoFisher, Carlsbad, CA, United States) at 1: 1000 dilution in blocking bufferat 4°C overnight. After three 10 min long TBST washes, the membrane was further incubated with a goat anti-rabbit secondary antibody conjugated to horseradish peroxidase (HRP) (catalog# NEF812001) (PerkinElmer, Shelton, CT, United States) at a dilution of 1: 10,000 in blocking solution for 1 hour. Three more TBST washes were performed for 10 min each. The protein bands were visualized using the Prometheus Prosignal Pico Reagent (Genesee Scientific, El Cajon, CA, United States) according to the manufacturer’s protocol on the ChemiDoc XRS Imaging System GAPDH was used as an internal loading control and detected with primary antibody mouse anti-human GAPDH (catalog# ab8245) (abcam, Boston, MA, United States) and secondary antibody goat anti-mouse IgG1 HRP (catalog# ab97240) (abcam, Boston, MA, United States) at a 1:10,000 dilution. The protein blots were visualized as above.

### 4.7 Generation of an immunodeficient MECP2 deficient mouse model

To generate the immunodeficient and *MECP2* deficient mouse model, we crossed the *Mecp2*^−/+^ heterozygous mice (JAX stock# 003890) with the *Rag2*^−/−^ homozygous mice (JAX stock# 008449) [29–31]. Heterozygous *MECP2* females (*Mecp2*^−/+^) and homozygous Rag2 males (*Rag2*^−/−^) were used for the crossings since homozygous *MECP2* females (*Mecp2*^−/+^) and hemizygous *MECP2* males (*Mecp2*^−/*y*^) are not viable past 10 weeks of age. The resulting immunodeficient mouse model, B6-Rag2^−/−^Mecp2^−/+^ (named BRM) were used for all of the *in vivo* studies. Genotyping was performed by PCR with the following primer sets according to the Jackson laboratory protocols listed for the respective genes: *MECP2* common 5′-AAATTGGGTTACACCGCTGA-3′, *MECP2* wild type 5′-CTGTATCCTTGGGTCAAGCTG-3′, *MECP2* mutant 5′-CCACCTAGCCTGCCTGTACT-3′, RAG2 wild type forward 5′-ATCAATGGTTCACCCCTTTG-3′, RAG2 wild type reverse 5′-TCATGTGAAAGCAGTTCAGGAC-3′, RAG2 mutant forward 5′-CCGCCATATGCATCCAAC-3′, RAG2 mutant reverse 5′-CAGCGCTCCTCCTGATACTC-3′.

All animals were housed in Techniplast cages (Techniplast, West Chester, PA, United States) in a temperature (68°F–72°F) and humidity (~25%) controlled vivarium maintained on a 12: 12 light-dark cycle. Mice were fed a standard diet of Teklad global 18% protein rodent diets 2918 (Envigo, Hayward, CA, United States). Rodent chow and tap water were available *ad libitum*. In addition to standard bedding, a Nestlet square, shredded brown paper, and a cardboard tube (Jonesville Corporation, Jonesville, MI) were provided in each cage.

### 4.8 Transplantation of BRM mice with human CD34+ HSPC

#### Adult mice:

Eight-week-old BRM (*Rag2*^−/−^*Mecp2*^−/+^) female mice (N = 4 per group) were conditioned by intraperitoneal injection of busulfan (20 mg/kg body weight) on days −2 and −1 pre-transplant. On day 0, mice were transplanted intravenously by tail vein injection with 500,000 total human CD34+ HSPC transduced with the EV or the *MECP2* vector. Wild type (WT) (B6-Rag2^−/−^ Mecp2^+/+^) mice were used as controls. The WT mice were used to evaluate any reversal or rescue of the phenotypes that result from the loss of MeCP2 toward WT levels of activity but were not transplanted with any cells and did not receive any busulfan conditioning.

#### Newborn mice:

Two-to-five-day old newborn BRM mice, regardless of sex, were conditioned with 125 rads using a Cesium source on the day of cell transplantation. Mice were subsequently transplanted intrahepatically with 500,000 total human CD34+ HSPC transduced with the EV or the *MECP2* vector. WT nontransplanted newborn mice were used as controls.

To allow for the transplanted cells to fully engraft, mice were left for 16 weeks post-transplant before being subjected to behavioral and motor testing by rotarod and open field.

### 4.9 Motor skill assays

#### Rotarod:

To evaluate motor coordination, grasping ability, and learning, transplanted mice were subjected to a rotarod assay. The mice (N = 4 per group for the newborn transplanted and N = 4 per group for the adult transplanted) were initially allowed to acclimate in the testing room for 1 hour. Mice were then placed on a rotating rod that gradually accelerated from 5 to 40 revolutions per minute over a 5-minute time span (Rotamex-5, Columbus Instruments, Columbus, OH, United States). Rotarod evaluation was performed on three consecutive days to assess motor learning. Latency to fall off the accelerating rod was scored manually with a maximum evaluation time of 2 min. If a mouse fell off the rod within 3 s of placement, a second trial was performed.

#### Open field assay:

To evaluate whether the *MECP2* vector transduced human CD34+ HSPC improved activity phenotypes associated with Rett syndrome, an open field assay was used to measure total activity, horizontal activity, and vertical rearing activity. The mice (N = 4 per group for the newborn transplanted and N = 4 per group for the adult transplanted) were first acclimated to the testing room for 1 hour. Mice were then subjected to a 15-minute open field assay in a 45 × 45 cm photocell-equipped Accuscan apparatus which automatically recorded the parameters.

To reduce carry-over effects from repeated behavioral testing, at least 24 h were allowed to pass between the completion of one task and the start of another. Assays were performed in the order of least to most stressful and between the hours of 8:00AM PST and 7:00PM PST during the light phase. Between subjects, testing apparatus surfaces were cleaned using 70% ethanol and allowed to dry.

For statistical analysis, the Student’s t-test was used to directly compare the *MECP2* mice to either the EV vector control mice to evaluate improvement in Rett syndrome phenotypes or to the WT mice to evaluate any reversion to WT levels. Statistical variance was similar between groups and data points for the adult and newborn transplanted mice. Personnel performing the rotarod and open field assays were blinded to the cohorts and only had access to the mouse identification numbers.

At the completion of the *in vivo* efficacy studies, the mice were euthanized and peripheral blood was collected and analyzed for lentiviral vector copy number by QPCR to determine the levels of engraftment of the gene modified cells, as described above. Brain tissue was also collected for evaluation of MeCP2 expression. All mouse handling and experiments were performed following the UC Davis IACUC policies. These experiments were designed and performed according to ARRIVE guidelines.

### 4.10 Immunohistochemistry

After the rotarod and open field assays were completed, the mice were euthanized, perfused with 4% paraformaldehyde, and the brain tissues were collected. Tissues were fixed in 4% paraformaldehyde and embedded in paraffin blocks. The blocks were sectioned in the sagittal direction at 5 μm directly onto SuperFrost slides. The slides were then stained using a Leica Bond automated immunostainer with a rabbit anti-mouse MeCP2 monoclonal antibody (catalog# ab253197) (abcam, Cambridge, UK) at a 1:8000 dilution in Citrate Epitope Retrieval buffer 1 at pH 6.0. A poly-HRP-IgG anti-rabbit secondary antibody (catalog# RE7280-CE) (Leica Biosystems, Deer Park, IL, United States) was then added. This was followed by DAB (3,3′-Diaminobenzidine) and hematoxylin used as counterstains. Images of the entire slides were then created using a Pannoramic SCAN (3D Histech). Enumeration of positive cells was then performed using the imageDx software. The Student’s t-test was used for statistical analyses to directly compare the *MECP2* tissue sections to either the control EV or the WT groups.

## Data Availability

The original contributions presented in the study are included in the article/supplementary material, further inquiries can be directed to the corresponding author.
